# Nature Engagement Outcomes of Viewing Nature Through a 360° Video or a Tablet Screen: Randomized Trial

**DOI:** 10.2196/63424

**Published:** 2025-06-16

**Authors:** Elena Brambilla, Karen Stendal, Vibeke Sundling, Giovanna Calogiuri

**Affiliations:** 1 Department of Nursing and Health Sciences, Faculty of Health and Social Sciences University of South-Eastern Norway Drammen Norway; 2 Department of Business, Marketing and Law, USN School of Business University of South-Eastern Norway Ringerike Norway; 3 Department of Optometry, Radiography and Lighting Design, Faculty of Health and Social Sciences University of South-Eastern Norway Kongsberg Norway

**Keywords:** virtual reality, immersive virtual nature, nature connectedness, nature engagement, nature-based interventions, outdoor, green exercise

## Abstract

**Background:**

Nature engagement, including affective and physical interactions with nature, is linked to a multitude of health and well-being benefits. Unfortunately, opportunities for engaging with nature are decreasing worldwide. University students, especially, are a demographic group that tends to engage little with nature. Immersive virtual nature (IVN; ie, digital nature content delivered through immersive devices, such as head-mounted displays) has been proposed as a medium to facilitate nature experiences and engagement. In recent years, 360° nature videos have emerged as an accessible way to create IVN content, although it is still unclear whether they can elicit presence and increase nature engagement to a greater extent than nature videos delivered through nonimmersive media.

**Objective:**

We aimed to investigate the effectiveness of nature videos as a medium to promote nature engagement among university students, comparing devices with different levels of immersion. Specifically, 2 experimental conditions were tested: a 360° nature video delivered through a head-mounted display (IVN) and a matching video displayed on a tablet screen (nonimmersive virtual nature).

**Methods:**

In total, 38 students were recruited at the library of a university campus and invited to participate in an organized hiking tour at the location displayed during the virtual nature experience. They were then randomized, using a random number generator, to either the IVN (n=20, 53%) or the nonimmersive virtual nature condition (n=18, 47%). Pre- and postexposure assessments of nature connectedness, intention to perform green exercise, intention to visit the hiking location, and intention to participate in the organized hiking tour were collected. Presence, cybersickness, and actual attendance on the tour were also assessed.

**Results:**

A mixed ANOVA showed statistically significant pre- to postexposure assessment increases in nature connectedness (*F*_1,36_=33.49; *P*<.001; η_p_^2^=0.48); intention to perform green exercise (*F*_1,36_=5.55; *P*=.02); intention to visit the hiking location (*F*_1,36_=15.34; *P*<.001; η_p_^2^=0.26); and intention to participate in the hiking tour (*F*_1,36_=12.45; *P*=.001; η_p_^2^=0.30). Both conditions were associated with medium to high ratings of “being there” and “sense of reality” but low ratings of “realism.” The cybersickness levels were generally low. Of the 38 students, 6 (16%) participated in the organized tour. The mixed ANOVA found no statistical differences between the two conditions for any of the outcomes. The participants’ changes in nature connectedness (ρ=0.35; *P*=.03) and attendance on the hiking tour (ρ=.37; *P*=.02) correlated with the presence item “being there.”

**Conclusions:**

This study provides novel evidence on the potential of virtual nature as a medium to improve nature engagement among university students, adding to the current debate on the effectiveness of 360° videos. These findings can inform future research as well as initiatives seeking to promote nature engagement.

## Introduction

### Background

The World Health Organization highlights the global need to foster mental health, reporting that 12% and 15% of the European and American populations experience psychological challenges, respectively [1]. College and university students are recognized to be a group at higher risk of mental health challenges, such as anxiety [2] and stress [3]. Recent reports show that the number of students reporting mental health issues has increased during the last decade from 11% to 31% [4]. Regular and recurrent nature engagement is a known salutogenic factor supporting the population’s general health and psychological well-being [5]. However, outdoor time is dropping, especially in younger generations [6], and college and university students often prefer exercising indoors (eg, in indoor gyms and sports facilities) rather than out in nature [4,7]. Meanwhile, the use of technology and screen time is rapidly increasing, contributing to further disconnection from nature [8,9]. From a different perspective, technology may be used as a tool to reconnect people to nature and improve human-nature interactions [10]. In this respect, virtual reality (VR) is gaining increasing attention due to its rapid advancement and economic accessibility for consumers [10,11].

### Benefits of Nature Engagement

According to the World Health Organization, direct exposure to nature (eg, contemplating the view of nature or spending time in, interacting with, or being physically active in a natural environment) provides mental and physical health benefits across socioeconomic strata and genders [12]. Emotional well-being, improved positive affect and mood, stress reduction, and social contact are just some of the benefits of human-nature interactions [13-15]. One important factor facilitating contact with nature and contributing to the health and well-being benefits associated with nature interactions is physical activity [16,17]. In this respect, Pretty et al [18] coined the concept of “green exercise,” referring to any physical activity in the presence of nature.

Not only physical contact with nature, but also cognitive and affective engagement with nature is linked to health and well-being outcomes [13,19-22]. As a relevant part of human-nature interactions, nature connectedness, referring to an individual’s sense of being emotionally connected to the natural world [23], plays an important role in human health. Studies link nature connectedness to various health outcomes including social, psychological, physical, and general well-being [19,24-26]. Moreover, evidence shows a positive correlation between nature connectedness and different well-being indicators, including happiness, mood, and life satisfaction, as well as reduced symptoms of stress-related mental disorders, and enhanced self-reported vitality, independently from a cultural and climatic context [20,27-29]. Nature connectedness can be classified as a personality trait or a temporary state [30]. However, the trait level of nature connectedness was found to be dynamic and varies across the life span [31,32].

It is important to highlight that, although being two different and largely independent ways of engaging with nature, a reciprocal influence exists between nature connectedness and physical contact with nature. Frequent experiences of nature or living near natural areas during childhood are important to strengthening people’s feelings about nature and fostering a trait level of nature connectedness [33-35]. Changes in temporary state levels of nature connectedness can be elicited in adults through direct contact with nature, including both passive and active engagement with nature, such as viewing nature while sitting in a park and brief walks in a forest [27,36,37]. A systematic review [36] suggests that interacting with nature on a regular basis can lead to sustained increases in nature connectedness. Furthermore, individuals with higher levels of nature connectedness are more likely to visit and spend time in a natural environment and engage in outdoor recreation [33,38]. Therefore, nature connectedness both supports and is supported by physical human-nature interactions [18,39,40]. Unfortunately, with more than half of the global population living in urban areas [41], the opportunities for human-nature interactions are decreasing worldwide [14]. The progressive disconnection from nature may have negative consequences on human health [14,42], including physical and mental health challenges, such as sedentary behavior, obesity, and reduced happiness and life satisfaction [43].

### Immersive Virtual Nature: The Case of 360° Videos

VR refers to a technology that provides the illusion of being in another place by substituting the primary sensory input with data generated by a computer [44]. VR can include different technologies with different levels of *immersion*, that is, “the extent to which the computer displays are capable of delivering an inclusive, extensive, surrounding and vivid illusion of reality to the senses of a human participant” [45]. For instance, head-mounted displays (HMDs), devices with a motion sensor that allow a 360° range of vision of a simulated environment while excluding the view of external surroundings, are commonly considered a form of immersive VR technology [46,47]. In contrast, computer screens are deemed as a form of nonimmersive technology. In this regard, the user’s sense of *presence* (ie, the subjective sense of being in the simulated environment [45]) has been indicated as a key factor in defining immersive experiences [45,48-53] capable of eliciting powerful psychophysiological responses [54]. While it is generally assumed that a user’s sense of presence is influenced by the levels of immersion of a given VR device, the extent to which immersive technology alone is sufficient to deliver an immersive experience, characterized by a strong feeling of presence, is still debated [50]. In this respect, *interactiveness* is considered paramount for delivering truly immersive VR experiences. Another factor is *cybersickness*, a specific type of visually induced motion sickness [55,56], which tends to be particularly problematic in highly immersive VR devices and can negatively impact the VR experience.

Immersive virtual nature (IVN) refers to installations that use immersive VR technology to provide an illusory perception of being surrounded by and interacting with a natural environment [11]. IVN has been found effective in eliciting increased nature engagement outcomes, including nature connectedness [57] and intention to visit naturalistic locations [58-61]. In recent years, 360° videos (spherical videos that can be viewed through HMD) emerged as an accessible technique to generate IVN contents [62]. However, concerns have been raised regarding the effectiveness of 360° videos compared with computer-generated interactive IVN experiences [57]. Moreover, the extent to which 360° nature videos can increase nature engagement to a greater extent than videos displayed through nonimmersive technologies (eg, viewing nature through pictures, videos, or different screens) is not clear [57]. While some studies indicate that 360° nature videos tend to be more effective in eliciting nature connectedness than traditional nature videos [47,63], others suggest less consistent results [64,65]. Moreover, to the best of our knowledge, to date, only a few studies have investigated the effects of 360° nature video experiences on people’s physical engagement with nature, including measurements of actual behavior, comparing experiences delivered through immersive versus nonimmersive technologies.

### This Study

The purpose of this study was to investigate the extent to which a single virtual nature experience could elicit increased nature engagement outcomes in university students, and whether this effect varies depending on the level of immersion of the devices through which they are delivered. Specifically, in this study, the IVN condition consisted of viewing a 360° video featuring a naturalistic location through an HMD, while the nonimmersive virtual nature (NIVN) condition consisted of viewing the same video in rectangular format on a tablet screen. In this study, nature engagement is used as an umbrella term to enclose affective (operationalized as nature connectedness) and physical interactions with nature (operationalized as behavioral intentions and actual behavior relative to nature visits).

The following research question was outlined: What is the effectiveness of viewing a video featuring a naturalistic location either through an immersive (IVN) or a nonimmersive device (NIVN) in promoting nature engagement among university students?

To address the research question, we proposed the following hypotheses:

1. There will be a significant pre- to postexposure assessment increment in all the nature engagement outcomes in both conditions.
2. Compared with the NIVN condition, the IVN condition will elicit larger increments of all nature engagement outcomes, as well as greater levels of presence and cybersickness.
3. All nature engagement outcomes will significantly and positively correlate with the participants’ level of presence and negatively correlate with the severity of cybersickness symptoms.

## Methods

### Research Design and Randomization

The study was designed as a randomized trial with parallel groups and pre- and postexposure assessments, and this paper is outlined following the CONSORT (Consolidated Standards of Reporting Trials) guidelines [66].

First, an ID code list was created, and each ID code was allocated to 1 of the 2 experimental conditions (IVN and NIVN) using a random number generator. Each participant was linked to a unique ID code just before undergoing the experiment. Anonymity was assured by storing participants’ identifying information (ie, name, family name, and phone number) only in paper form separately from the collected data and by deleting it after completion of the project. The allocation sequence was concealed until participants were enrolled and assigned to the intervention. However, the trial was not completely blinded, as the participants were aware of the two experimental conditions.

### Participants and Sample Size

The study took place from March 16, 2022, to April 28, 2022, at the library of the University of South-Eastern Norway, which offers bachelor and master programs in nursing, computer sciences, teacher education, management, and political sciences. University students were purposely targeted as a specific group of interest, as they are a population that tends to prefer indoor activities [4] and engage less in outdoor recreation [33]. Therefore, they could particularly benefit from exposure to the virtual nature. Participants were recruited through flyer distribution, advertisements posted on social media, and presentations of the study during classes. Eligible participants were university students living in Drammen, speaking Norwegian or English, and able to walk outdoors without aid. No other restrictions were applied. Participants were informed in verbal and written form about the general purpose of the study and its associated risks.

The sample size was estimated based on the primary outcome, nature connectedness. Two studies [47,67] investigating the effects of experiencing an IVN found a large and a medium effect size for nature connectedness. Hence, for this study, a medium effect size was selected to ensure sufficient power to detect meaningful effects without unnecessarily burdening a larger number of participants. The sample size calculation was performed in G*Power (Heinrich Heine University) for mixed between-within subject ANOVA with 2 groups and 2 repeated measurements, setting an expected power of 0.80, a precision of 0.05, and a moderate effect size (*f*=0.25), which resulted in an optimal sample size of 34. In total, 54 participants were recruited and assessed for eligibility, of whom 7 did not meet the eligibility criteria. The other 9 participants were enrolled but had to be excluded because of technical issues (ie, malfunctioning of the equipment). The final sample (participants included in the analyses) consisted of 38 participants.

### Virtual Nature Experiences

The IVN condition consisted of a 360° video of nature, delivered through a stand-alone HMD (Meta Quest 2, Reality Labs). Participants viewed the 360° video while sitting on a chair. In the NIVN condition, participants watched the same video on a tablet screen (a 26.7 cm, full high-definition, 1200-pixel LCD). The version of the video used in either condition was equal in duration. The video was filmed in a local forest named Dronningslepet and composed of a sequence of static clips (15 seconds each). The total length of the video was 5 minutes, which is generally deemed sufficient to elicit psychological responses while reducing the risk of cybersickness [10,57]. The video was filmed using a GoPro MAX (3840×2160 resolution; 25 frames per second) and edited in DaVinci Resolve 18 (Blackmagic Design) using sounds retrieved from the YouTube Audio Library [68]. A demo of the video is available on the web [69].

### Procedures

Before undergoing either the IVN or the NIVN condition, participants were informed about the safe use of the technology. Subsequently, participants were invited to join in an organized hiking tour taking place at the same location where the video, displayed during the experiment, was filmed (ie, a local forest named Dronningslepet). Information regarding the tour duration, path length, and intensity was provided both verbally and in written form. Within 5 weeks from the experiment, participants could attend the tour (3 dates were made available), which had a duration of approximately 3 hours and included a local guide, food, and transportation. The participants were then asked to fill in the pre-exposure questionnaire.

**Figure 1:**
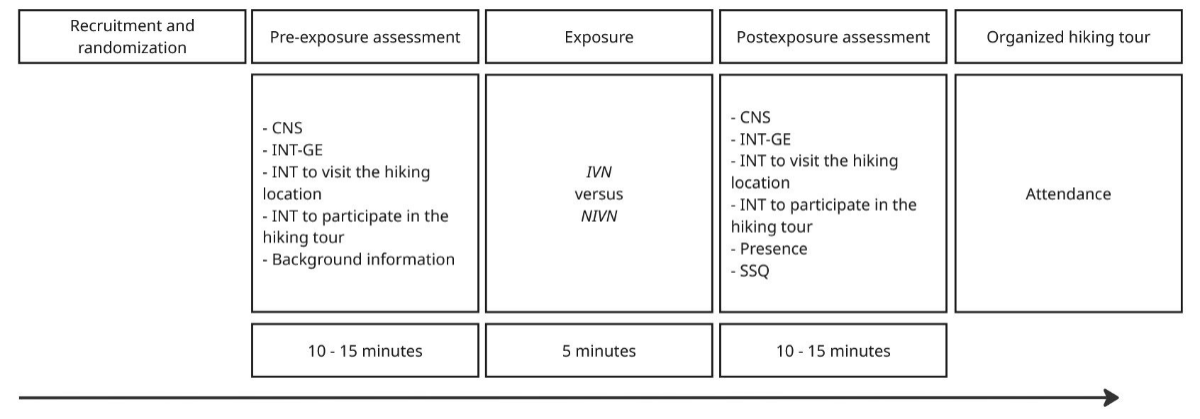
Experimental procedure, data gathering organization, and procedure duration. Background information included age, gender, faculty, residence, green exercise habits, and previous visits to the hiking location. CNS: Connectedness to Nature Scale; INT: intention; INT-GE: Intention to Perform Green Exercise questionnaire; IVN: immersive virtual nature; NIVN: nonimmersive virtual nature; SSQ: Simulator Sickness Questionnaire.

At the completion of each experimental condition, the participants filled in the postexposure questionnaire. A reminder about the tour was sent to them via phone before each date. The participants who were allocated to the NIVN condition were invited to try the IVN condition after all the hiking tours were completed. The total duration of the procedure varied from 20 to 35 minutes. The experimental procedure is summarized in Figure 1. Data collections were handled by questionnaires created with the Nettskjema, a web-based survey solution developed and hosted by the University of Oslo. The questionnaires were provided in Norwegian and English to allow both international and local students to participate.

### Outcomes

#### Nature Engagement Outcomes

##### Overview

The main outcomes included self-reported nature engagement collected by questionnaire before and after each experimental condition. In addition, attendance on the organized hiking tour was recorded. The questionnaires were available in both English and Norwegian. The English versions of the questionnaires were all validated. The questionnaires were translated from English to Norwegian by native speakers in collaboration with academics with expertise in environmental psychology, ensuring consistency with the cultural context.

The main outcomes related to both affective and physical engagement with nature and operationalized through the concept of nature connectedness, intention to visit nature, and actual visitation of nature. In total, 3 types of nature visits were considered: generic green exercise, independent visit to the location of the hiking tour (behavioral intentions), and participation in the organized hiking tour (behavioral intention and actual behavior). The questionnaires are provided in Multimedia Appendix 1.

##### Nature Connectedness

The state version of the Connectedness to Nature Scale (CNS) [27] was used to assess the extent to which individuals felt emotionally attached to the natural world. CNS was selected for its comprehensive assessment of the relationship between humans and nature (including animals, plants, and a sense of equality between the self and nature) and its high reliability [27]. The CNS demonstrated strong internal consistency, as measured by Cronbach α (α=0.82), which is consistent with previous studies (α=0.80–0.82) [27,67]. Additionally, the CNS was selected for its convergent validity and its positive correlation with other measures of nature connection such as the Inclusion of Nature in Self scale [70]. The scale includes 13 items (eg, “Right now I’m feeling a sense of oneness with the natural world around me”), rated on a 7-point Likert scale (1=strongly disagree; 7=strongly agree). The internal consistency of the scale in this study, as measured by Cronbach α (α=0.81-0.85), was consistent with previous studies [27,67].

##### Intention to Perform Green Exercise

The Intention to Perform Green Exercise questionnaire (INT-GE) [71], assessing the extent of intention to engage in green exercise in the near future, was used. The INT-GE has demonstrated a strong factorial validity in the original validation study [71] and both the original English version [71] and translated Norwegian version [58] demonstrate good internal consistency, as measured by Cronbach α (α=0.94-0.95). The scale includes 5 items (eg, “I expect to do Green Exercise”), rated on a 7-point Likert scale (1=strongly disagree or very unlikely; 7=strongly agree or very likely). Examples of what was intended as “green exercise” were provided in the caption, which also provided a time reference (“over the next 5 weeks”), that approximately corresponded to the time span between the participant’s virtual nature experience and the last planned hiking tour. In this study, the internal consistency of the scale, as measured by Cronbach α (α=0.95-0.95), was in line with previous studies [58,72].

##### Intention to Visit the Hiking Location

This scale was developed following established guidelines for developing questionnaires based on the theory of planned behavior within health services researchers [73]. Specifically, the participants’ intention to visit the hiking location was assessed through 3 items (eg, “After I received information about Dronningslepet, I consider visiting it within the next 5 weeks”) rated on a 7-point Likert scale (1=strongly disagree; 7=strongly agree). The scale in this study showed an adequate internal consistency, as measured by Cronbach α (α=0.93-0.97).

##### Intention to Participate and Actual Participation in the Organized Hiking Tour

A single-item question about the participants’ intention to attend the organized hiking tour (ie, “After I received information about the organized trip to Dronningslepet, the probability that I will participate in the tour is...”), rated on an 11-point Likert scale (0=very low; 5=neither high or low; 10=very high), was used. In addition, the participants’ attendance on the organized hiking tour was registered in loco by one author.

#### Other Outcomes

##### Presence

Postexposure assessment of the sense of presence was performed based on the study by Slater et al [74], with some adjustments relative to the context of the trial and consistent with previous work by Calogiuri et al [75]. The final 3 items were rated on an 11-point Likert scale (0=absolutely disagree; 10=absolutely agree) and formulated as follows: (1) “In the video I had the sense of ‘being there’” (ie, “being there”); (2) “I thought of the video as equal to the real environment” (ie, “realism”); and (3) “The video became more real or present to me compared to the real world; by “real world” we mean the room where you were undergoing the test (ie, “sense of reality”). In line with previous works [75,76], the items were used individually for further analysis.

##### Cybersickness

The Simulator Sickness Questionnaire (SSQ) [77] was administered after both experimental conditions to measure the severity of cybersickness. The SSQ has demonstrated good factorial validity in the original validation study [77]. The SSQ includes 16 items describing different symptoms (eg, “nausea,” “headache,” and “eyestrain”) and rated with a 4-point Likert scale (0=not at all; 4=a lot). The scale in this study showed adequate internal consistency, as measured by Cronbach α (α=0.86), consistent with a previous study that used the same Norwegian version of the scale [78].

##### Background Information

Participants’ age, gender, and residence details were collected for descriptive purposes. Furthermore, previous visits to the hiking location (ie, “Have you been to the hiking location before?”) were assessed through a single-item question to which participants could answer “yes,” “no,” or “I don’t remember.” In addition, green exercise habits (ie, *“*How often do you do green exercise?”) were assessed through a single-item question to which participants could answer “more than 3 times a week,” “2 times a week,” “1 time a week,” or “less than 1 time a week.”

### Statistical Analysis

The score of all instruments was computed in line with instructions provided by the respective authors [27,71,73,74,77,79]. Reliability analysis was performed to control the scales’ internal consistency. To detect possible missing values and outliers and estimate the variables’ frequency distribution, explorative analysis was conducted. No missing or extreme values were identified except for INT-GE values, which included one outlier that was detected and adjusted, in line with Tabachnick and Fidell [80], by replacing the outlier’s score with a raw score of one unit smaller than the most extreme score in the distribution. This approach avoided the score distorting the statistic while maintaining the outlier value deviant but not as deviant as it was [81]. The frequency distributions were explored based on the examination of histograms and values for skewness at both time points, showing an acceptable normal distribution for all the variables and moderately skewed distribution for INT-GE. The skewness values for pre and postexposure assessment were as follows: CNS=0.03 and −0.02; INT-GE=−0.99 and −0.95; intention to participate in the hiking tour=0.43 and −0.35; and intention to visit the hiking location=−0.34 and −0.87.

As data followed normal distribution and variances were homogenous, a parametric mixed ANOVA was conducted to assess the impact of the two experimental conditions (between-subjects effect) on the dependent variables (ie, CNS, INT-GE, intention to visit the hiking location, and intention to participate in the hiking tour) across the 2 time points (within-subjects effect). The within-subjects factor was “time point” (pre- and postexposure assessment) while “condition” (IVN or NIVN) was set as the between-subjects factor. The effect size was expressed as partial eta squared (η_p_^2^). A possible difference between the conditions for the presence items and the SSQ score were tested through a Mann-Whitney *U* test. Spearman rank correlation coefficient (ρ) was used to explore possible associations among all variables. For the variables with repeated measurements, differential scores from pre- to postexposure assessment (delta values) were computed and used in the correlation analysis. The strength of the correlation was interpreted according to the guidelines proposed by Cohen [82].

All analyses were conducted using SPSS (version 28.0.0.0.1; IBM Corp).

### Ethical Considerations

A preliminary assessment by the Norwegian Regional Committees for Medical and Health Research Ethics determined that, in accordance with the Norwegian Health Research Act, the study did not require a full evaluation, as it did not involve medical or health interventions, the collection of sensitive personal data, or research on vulnerable populations. Accordingly, this randomized trial was not registered on a randomized controlled trial repository at the time of initiation. Subsequently, the trial was registered at the Norwegian Centre for Research Data, presently known as the Norwegian Agency for Shared Services in Education and Research (registration number 185017). Informed consent was obtained and delivered in written form both in English and Norwegian languages. It included information concerning the purpose of the study, contact details of both the project leader and the institution’s data protection officer, legal basis for processing data, voluntary participation and the possibility to withdraw without having to give any reason for doing so, and anonymity. The data collected for the study have been deidentified to ensure participant confidentiality and privacy. A lottery prize consisting of an NOK 500 (US $55) gift card was offered. The study was conducted in compliance with the Guidelines for Research Ethics in the Social Sciences and the Humanities [83].

## Results

**Figure 2:**
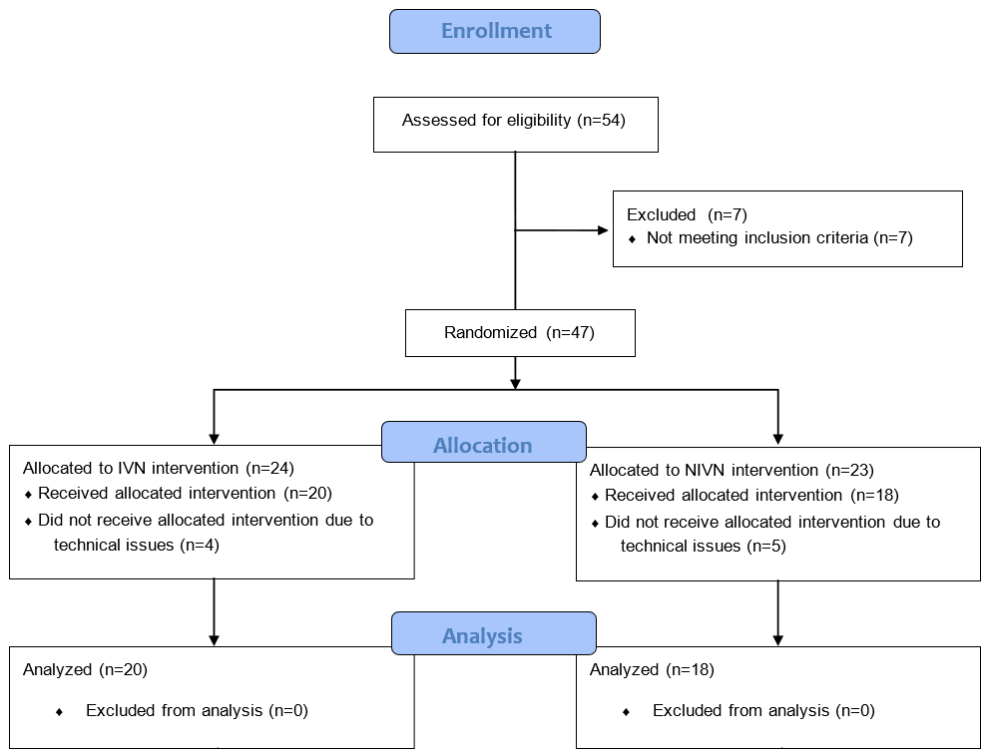
CONSORT flow diagram of the study phases concerning participant enrollment, allocation, and analysis. IVN: immersive virtual nature; NIVN: nonimmersive virtual nature.

A flow diagram of the experimental procedure and data collection process through the study phases is presented in Figure 2 [66].

### Hypothesis 1: Changes in Nature Engagement

The mixed between-within subjects ANOVA (Table 1) showed a significant pre- to postexposure assessment increase in both conditions, with a moderate to large effect size for INT-GE (Wilks Λ=0.87; *F*_1,36_=5.55; *P*=.02; η_p_^2^=0.13) and a large effect size for CNS (Wilks Λ=0.52; *F*_1,36_=33.49; *P*<.001; η_p_^2^=0.48); intention to visit the hiking location (Wilks Λ=0.70; *F*_1,36_=15.34; *P*<.001; η_p_^2^=0.30); and intention to participate in the hiking tour (Wilks Λ=0.74; *F*_1,36_=12.450; *P*=.001; η_p_^2^=0.26).

**Table 1:** Descriptive results of pre and postexposure assessment of the main outcomes (N=38) and mixed between-within ANOVA.

Items			NIVN^a^ (n=18), mean (SD)		IVN^b^ (n=20), mean (SD)		Main effect of time					Time and condition interaction					Effect of condition		
							*F* test (*df*)		*P* value		*F* test (*df*)			*P* value		*F* test (*df*)			*P* value
**CNS^c^**							33.49 (1,36)		<.001		0.14 (1,36)			.71		0.85 (1,36)			.36
	Before	4.65 (1.09)		4.42 (0.78)															
	After	5.18 (1.04)		5.18 (1.04)															
**INT-GE^d^**							5.55 (1,36)		.02		0.31 (1,36)			.37		0.78 (1,36)			.38
	Before	5.98 (1.20)		5.60 (1.66)															
	After	6.24 (0.96)		5.79 (1.58)															
**Intention to visit the hiking location**							15.34 (1,36)		<.001		1.08 (1,36)			.31		0.06 (1,36)			.94
	Before	4.48 (1.44)		4.28 (1.84)															
	Before	5.07 (1.84)		5.02 (2.09)															
**Intention to participate in the hiking tour**							12.45 (1,36)		<.001		0.02 (1,36)			.89		0.55 (1,36)			.46
	Before	6.72 (2.24)		6.25 (1.65)															
	After	7.56 (1.82)		7.56 (1.82)															

^a^NIVN: nonimmersive virtual nature.

^b^IVN: immersive virtual nature.

^c^CNS: Connectedness to Nature Scale.

^d^INT-GE: Intention to Perform Green Exercise questionnaire.

### Hypothesis 2: Differences Between Experimental Conditions

No statistically significant difference between baseline levels of the experimental conditions emerged for nature engagement outcomes and participants’ characteristics (Table 2). No statistically significant condition by time point on effect was found for any of the main outcomes, indicating no difference in the pre- to postexposure assessment changes of the outcomes between the experimental conditions (Table 2). Of the 38 participants, 6 (16%) attended the hiking tour, 3 from each experimental condition (IVN: 3/20, 15% and NIVN: 3/18, 17%; possible differences between the conditions were not tested statistically). The Mann-Whitney U test for the items of presence (ie, “being there,” “sense of reality,” and “realism”) revealed no statistically significant difference between the IVN and the NIVN conditions (Table 3). In total 35% (7/20) of participants in the IVN condition and 44% (8/18) of participants in the NIVN condition showed an SSQ total score higher than 20, which indicates severe symptoms of cybersickness, although the median values of the SSQ total score levels did not exceed the score of 20. The Mann-Whitney *U* test found no statistically significant differences between the experimental conditions for cybersickness.

**Table 2:** Participants’ characteristics and evaluation of groups’ baseline differences (N=38).

Characteristics				NIVN^a^ (n=18)		IVN^b^ (n=20)		Between-groups tests		*P* value
Age (y), mean (SD)				24.94 (7.07)		24.55 (3.41)		*U=*146.50		.33
**Gender,** **n (%)**								χ^2^_1_=0.05; φ=−0.09		.82
		Men	7 (39)		6 (30)					
		Women	11 (61)		14 (70)					
		Nonbinary	0 (0)		0 (0)					
CNS^c^, mean (SD)				4.65 (1.09)		4.42 (0.78)		Mean difference=−0.24 (95% CI −0.86 to 0.38); *t*_38_=−0.78		.44
INT-GE^d^, mean (SD)				5.98 (1.20)		5.60 (1.66)		Mean difference=−0.33 (95% CI −1.15 to 0.50); *t*_38_=−0.80		.64
Intention to visit the hiking location, mean (SD)				4.48 (1.44)		4.28 (1.84)		Mean difference=−0.20 (95% CI −1.29 to 0.90); *t*_38_=−0.37		.43
Intention to participate in the hiking tour, mean (SD)				6.72 (2.24)		6.25 (1.65)		Mean difference=−0.47 (95% CI −1.75 to 0.81); *t*_38_=−0.74		.08
**Previous visits to the hiking location, n (%)**								χ^2^_2_=0.44; φ=0.11		.80
	Yes		2 (11)		3 (15)					
	No		13 (72)		15 (75)					
	I do not remember		3 (17)		2 (10)					
**Green exercise routine, n (%)**								χ^2^_1_=0.66; φ=0.19		.42
	Two times a week or more		10 (56)		8 (40)					
	One time a week or less		7 (39)		12 (60)					

^a^NIVN: nonimmersive virtual nature.

^b^IVN: immersive virtual nature.

^c^CNS: Connectedness to Nature Scale.

^d^INT-GE: Intention to Perform Green Exercise questionnaire.

**Table 3:** Descriptive results of items of presence and cybersickness (N=38) stratified by groups and Mann-Whitney U test results.

Items	NIVN^a^, median (IQR)	IVN^b^, median (IQR)	Mann-Whitney *U* test	*P* value
Being there	8.00 (6.75-9.00)	7.50 (6.00-8.75)	203.00	.52
Realism	5.50 (1.75-7.00)	3.50 (1.50-4.75)	237.000	.10
Sense of reality	6.00 (3.00-8.00)	7.00 (5.25-8.75)	130.000	.15
SSQ^c^ score	18.70 (7.48-46.75)	14.96 (4.67-33.66)	198.000	.60

^a^NIVN: nonimmersive virtual nature.

^b^IVN: immersive virtual nature.

^c^SSQ: Simulator Sickness Questionnaire.

### Hypothesis 3: Association of Nature Engagement With Presence and Cybersickness

The correlational analysis performed among all outcomes from both IVN and NIVN conditions is presented in Table 4. The presence component “being there” showed a significant moderate and positive correlation with CNS (ρ=0.33; *P*=.03) and participation in the hiking tour (ρ=0.37; *P*=.02). However, it is important to stress that the validity of “being there” with participation in the hiking tour was limited by the small number of participants attending the hiking tour, hence caution is warranted in interpreting this outcome. Other significant correlations emerged among the indicators of nature engagement, specifically between intention to participate in the hiking tour and intention to visit the hiking location (ρ=0.39; *P*=.02) and between intention to visit the hiking location and INT-GE (ρ=0.34; *P*=.04). No other significant correlation emerged from the analysis.

**Table 4:** Spearman rank correlation coefficient of indicators of nature engagement in immersive virtual nature (IVN) and nonimmersive virtual nature (NIVN) conditions, expressed as differential scores from pre- to postexposure assessment (delta values), with presence and cybersickness (N=38).

Indicators of engagement with nature (delta values)		Being there	Realism	Sense of reality	SSQ^a^ score	1	2	3	4
**1. CNS^b^**									
	ρ	0.35	0.30	0.11	–0.21	—^c^	—	—	—
	*P* value	.03^a^	.06	.51	.21	—	—	—	—
**2. INT-GE^d^**									
	ρ	0.09	0.09	–0.20	–0.02	–0.14	—	—	—
	*P* value	.58	.58	.23	.88	.41	—	—	—
**3. Intention to visit** **the hiking location **									
	ρ	0.29	0.14	0.28	0.00	0.04	0.34	—	—
	*P* value	.07	.40	.08	.99	.79	.04^e^	—	—
**4. Intention to participate in the hiking tour **									
	ρ	0.21	0.31	0.22	0.24	0.07	0.25	0.39	—
	*P* value	.21	.06	.19	.15	.70	.13	.02^e^	—
**5. Participation in the hiking tour**									
	ρ	0.37	0.03	0.12	–0.23	0.23	0.29	0.06	0.23
	*P* value	.02^a^	.87	.46	.16	.17	.12	.57	.71

^a^SSQ: Simulator Sickness Questionnaire.

^b^CNS: Connectedness to Nature Scale.

^c^Not applicable.

^d^INT-GE: Intention to Perform Green Exercise questionnaire.

^e^Correlation is significant at the .05 level (2-tailed test).

## Discussion

### Principal Findings

University students are often at a high risk for poor mental health [2-4] and tend to engage little with nature [4,7,33]. This randomized trial investigated the effectiveness of a single IVN or NIVN exposure in eliciting nature engagement among university students, examining whether the former can provide better effects compared to the latter. Possible associations of nature engagement outcomes with the participants’ feelings of presence and cybersickness were also investigated. The findings showed an overall pre- to postexposure assessment increase in nature engagement outcomes for both conditions. Compared to NIVN, IVN did not provide additional benefits on nature engagement nor did it increase the likelihood of participation in an organized hiking tour. Furthermore, no statistically significant differences emerged between IVN and NIVN conditions concerning presence or cybersickness. Nature connectedness and participation in the hiking tour positively correlated with the presence item “being there.” No significant correlation was found among any of the nature engagement outcomes and the other items of presence or cybersickness.

### Promoting Nature Engagement Through Virtual Nature

Supporting prediction (hypothesis 1), when compared with pre-exposure, postexposure levels of nature engagement were significantly higher in both the IVN and the NIVN conditions. These results are largely in line with previous systematic reviews investigating the effects of direct and indirect contact with nature connectedness [36,57]. Different indirect nature exposure through audio-visual presentation devices, such as HMDs, computer screens, and nature images, can provide opportunities for fostering a person’s sense of nature connection [10,36,47]. Furthermore, this study supports the previous research showing the effectiveness of virtual nature in fostering nature connectedness [27,36,84-86] and provides novel insights concerning the effectiveness of virtual nature as a tool for the promotion of physical engagement with nature, which has until now received little research attention [58].

Although the improvements observed in this study may seem relatively small, it should be noted that this is in line with previous studies [36,58,67], the effect size was moderate to large for INT-GE and large for the remaining outcomes. Furthermore, the average increment observed after just a single IVN exposure was considerable: 4% for INT-GE, 6% for CNS, 7% for the intention to visit the hiking location, and 8% for the intention to participate in the hiking tour. This suggests that even a short and relatively simple virtual nature experience holds practical relevance in the pursuit of promoting nature engagement. As indicated by a previous systematic review [36], these outcomes may have long-term implications, especially when virtual nature experiences are repeatedly proposed over time. Moreover, the increased nature engagement elicited by the virtual nature experience may lead to more frequent contact with nature, thereby yielding additional long-term physical and mental health benefits. In this respect, although the actual participation in the hiking tour was low (6/38, 16% participants and 6/28, 21% participants who reported a high intention to participate in the organized hiking tour in the postexposure assessments), we cannot exclude that the virtual nature exposure may have elicited other forms of nature visits (eg, green exercise and independent visits to other naturalistic locations). Indeed, while participation in the hiking tour was bound to a relatively rigid time schedule, time flexibility and convenience are known to be important factors sustaining green exercise and frequent nature visits [87].

### Immersive Versus Nonimmersive Experience?

Contrary to prediction (hypothesis 2), no statistical difference between the two conditions in the extent to which they elicited enhanced nature engagement outcomes emerged. This finding partly aligns with previous literature, although the evidence regarding this topic is still limited and largely mixed [57,88]. Indeed, while some studies suggest that IVN tends to elicit increased levels of nature connectedness to a greater extent than NIVN [47,63], other studies show no statistical differences [64,89]. While the findings of our study support the notion that immersion in itself has little or no impact on the nature engagement outcomes, other factors (eg, technology used, type of IVN scenarios, and degree of interactivity) may contribute to explaining the inconsistency of the findings in this field [10,47,58,90]. Therefore, it is important to stress the fact that our IVN condition was unable to generate greater feelings of presence compared with the NIVN condition. This may be explained by various factors: the relatively short duration of the 360° video, the lack of interactivity, the fact that the HMD was not paired with a supplementary high-quality headphone, and the low image resolution generally associated with consumer-oriented HMDs, especially if displaying 360° videos. Such an assumption finds support in the literature. For instance, a recent systematic review [57] investigating the effects of IVN on nature connectedness found consistent difference between IVN and NIVN only in studies using computer-generated scenarios (ie, computer graphics digitally recreating fictional or actual environments) but not among studies involving 360° videos. Moreover, it has been suggested that the levels of interactivity, as well as the quality and capability of the hardware used, as technological characteristics that enhance immersion, also play an important role in the extent to which IVN can effectively promote nature visits [58,67].

These findings prompt 2 reflections relative to the complex nature of immersion and the effectiveness of consumer-oriented VR technology. First, immersion is a fluid and complex construct [91], and despite that the levels of immersion are often linked to characteristics of the hardware such as frame rate and image resolution, it has been pointed out that software also plays a role in determining the immersion level [49]. Hence, having a 360° field of view and excluding the view of the real world may not be the sole technical aspect necessary to achieve a high level of immersion in IVN exposures. Second, consumer-oriented technology entails some limitations, especially regarding its inferior quality and capacity compared to state-of-the-art VR technology. Nevertheless, consumer-oriented technology bears practical advantages. For instance, 360° videos are often highly realistic, relatively easy to produce, and cost-effective. This is especially useful for replicating local natural environments in real-world promotional initiatives. Furthermore, VR technology represents an attractive form of entertainment for many individuals, with benefits in terms of public engagement. Hence, we envisage that with the ongoing improvement in the quality and capacity of consumer-oriented VR technology, its effectiveness in research and health-promotion initiatives will also increase.

### Presence, More Than Immersion, as a Key Factor for Nature Engagement Promotion

Partially supporting hypothesis 3, nature connectedness and participation to the hiking tour were associated with the presence item “being there.” These findings suggest that, beyond the hardware used (HMD or tablet screen), disturbances (eg, cybersickness), and perceived fidelity of the scenario, the subjective sense of “being there” may be a pivotal element in linking virtual nature experience and nature engagement. It is important to stress that the validity of these correlations is limited by the small sample size, especially as only 6 participants attended the hiking tour. Nevertheless, this finding aligns with previous research. For instance, a recent study [89] showed that, while exposure to IVN did not enhance higher levels of nature connectedness compared to its NIVN counterpart, participants who experienced a higher sense of embodiment in the virtual world tended to reflect more about nature and their role toward it. Furthermore, other studies also found that “being there,” more than other aspects of presence, positively correlated with different psychological outcomes [10,75,79,92]. Indeed, this item (ie, in the computer-generated world I had the sense of “being there”), is considered a core component of presence [45], distinct from, although related to, “realism” (ie, the extent to which the participant perceived the virtual world as similar to the real world) and “sense of reality” (ie, the extent to which the virtual world became more present than the real world).

However, questions remain on how the feeling of “being there” may facilitate enhanced nature connectedness and motivation to visit nature. It has been suggested that anticipated psychological benefits [58] and conditioned responses [93] may play an important role in this respect. In line with such perspectives, a vivid sensation of “being in” a natural environment would trigger the recall of past experiences, alongside the psychological states associated with it. This would, in turn, foster positive attitudes toward nature as well as a desire of reenacting those positive experiences.

It is important to emphasize that a person’s sense of presence can be influenced by several subjective factors, regardless of the technology used to deliver a VR experience. Indeed, immersion and presence are interrelated but distinct concepts and, while it is believed that the former is a primary determinant of the latter [94], their relationship is complex. As the feeling of presence is subjective and can, among other things, depend on individual personality traits [95], the exposure to the same type of technology may evoke varying levels of presence among different individuals. Hence, as highlighted by the findings of this study, some individuals may experience high levels of presence in NIVN exposures—although it is also important to consider the limitations of the proposed IVN condition and the extent to which it may be actually considered a fully immersive VR installation.

Cybersickness did not significantly correlate with measurements of nature engagement. However, it should be noted that the IVN condition did not generate noteworthy challenges regarding cybersickness. This is supported by the fact that, despite some participants reporting “severe” symptoms of cybersickness according to the cut-off provided by Kennedy et al [77], the median values for the SSQ score were considerably lower when compared with values reported in previous literature. Indeed, a systematic review [96] reported a pooled total SSQ mean score of 28 across 55 studies for VR experiences delivered through HMDs. Moreover, no significant difference in cybersickness symptoms was found between the IVN and the NIVN, indicating that the reported symptoms may be associated with pre-existing states. Indeed, several cybersickness symptoms, such as headache, fatigue, and difficulty focusing and concentrating, may be commonly experienced in daily life.

### Strengths and Limitations

#### Overview

One of the strengths of this study lies in the novelty of investigating the effects of IVN compared with NIVN on outcomes of physical engagement with nature, both regarding behavioral intention and actual behavior. As highlighted by two recent systematic reviews [57,88], studies on this specific topic are sparse, and further evidence on nature connectedness outcomes is needed. Moreover, to the best of our knowledge, this is the only randomized study that compared the effects of IVN versus NIVN on such outcomes, exploring factors that may influence the effectiveness of IVN, such as presence and cybersickness. Furthermore, the paper is outlined following the CONSORT guidelines [97]. However, some limitations need to be considered.

#### Limitations Relative to Design

First, the lack of a control group (ie, without virtual stimulation) limits the extent to which this study can establish a causal relationship between the experimental conditions and the outcomes. Second, the proposed IVN condition, consisting of a 360° video delivered through a stand-alone HMD, may not be considered fully immersive. Different findings may have emerged if a different technology (eg, including a high-end HMD or an interactive experience) was used.

#### Limitations Relative to the Sample

Different factors may have influenced the results regarding the participation in green exercise and the organized hiking tour. For instance, we purposefully targeted a population that tends to prefer indoor activities. Moreover, the study was conducted during the academic semester and examination sessions. While this strengthens the ecologic validity of the study, the tour dates may have conflicted with participants’ study-related commitments. In addition, the demanding intensity of the tour (approximately 3 hours duration) may have been an impediment for some participants. Information regarding the participants’ course of study was not recorded. However, we know that the students dwelling on the campus are largely linked to courses in nursing, computer sciences, teacher education, management, and political sciences—the campus does not offer courses in sports sciences or outdoor recreation. The participants may have had a personal interest in nature-based activities, which we were able to compute through the preassessments. Nevertheless, this could still be a potential source of bias, as the individual’s interest in nature may have influenced both attendance and responses to the study.

The sample size calculation was based on an expected medium effect size, as in line with previous studies [47,67]. However, these studies used different VR technology, which succeeded in eliciting stronger experiences of presence, possibly leading to larger changes in nature connectedness. Hence, the sample size calculation for our study may have been underpowered. In addition, it should be noted that the sample size calculation was performed based on nature connectedness and a mixed between-within subjects ANOVA (required to address hypotheses 1 and 2); hence, the sample may be underpowered for the other outcome variables and for the correlation analysis.

#### Limitations Relative to the Instruments

The CNS, INT-GE, and SSQ, as well as the presence items, were previously validated through psychometric studies and are generally accepted as valid instruments. The Norwegian translations of these scales were used in previous similar research [58,67], showing adequate internal consistency and patterns similar to their original counterparts. However, the Norwegian translations have not been validated through comprehensive psychometric investigations. Relative to the use of intention-based measurements, it should be noted that meta-analysis evidence found a significant, although weak, association between intention and behavior in various contexts [98-100]. This highlights the value that such assessment may hold in research focused on health- or well-being–related behavior. However, the measure of intention to visit the hiking location was developed based on established guidelines [73] but not validated through comprehensive psychometric investigations. Hence, caution is warranted when interpreting the findings relative to these outcomes. These instruments also showed very high Cronbach α values, which may indicate redundancy among items [101]. Finally, some considerations should be made regarding the cultural context of this study. In Norway, there is an abundance of natural environments, and this may have impacted the results of the study concerning intentions to visit nature.

### Implications for Practice and Future Research

These findings have significant implications in public and private institutions and organizations’ initiatives seeking to promote nature engagement among university students. Given the health benefits associated with nature engagement and the progressive disconnection from nature among young adults [14,42], schools, universities, municipalities, and health-promoting organizations may use virtual nature interventions, either in the form of low-end IVN or NIVN, as a tool in health-promoting and environmental education initiatives. Additional practical implications involve using virtual nature in interventions and initiatives across diverse contexts and population groups. For instance, urban citizens also often experience challenges accessing nature daily and may particularly benefit from virtual nature interventions. Businesses may also use virtual nature as a health-promotion activity to foster nature engagement among employees as an adaptive coping strategy. The use of virtual nature in such contexts and target groups should be further investigated.

The evidence on the effects of virtual nature on physical engagement with nature is still in its infancy and warrants more research. This should be appraised through either follow-up assessments measuring participants’ frequency of nature visitation or proposing opportunities for in-real-life nature experiences. Recommendation for future research includes comparisons of multiple experimental conditions and possibly a control condition. For instance, highly immersive VR technologies, such as high-end VR systems and interactive IVN experiences, should be considered as worthy of investigation. In addition, potential seasonal variations in nature engagement could be explored by diversified virtual nature environments. Moreover, while the ecologic validity of the study lies in it being conducted during the examination sessions, exploring the effects of virtual nature environments in different academic semesters, where students have varying study-related commitments, may also be worth investigating. Furthermore, little is known about repeated exposures and follow-up assessments exploring the longevity and stability of the acute change in nature engagement following virtual nature exposure. Given the rapid development of VR technology, further investigation may use or compare different hardware and software.

### Conclusions

This study shows that a single exposure to a 5-minute 360° nature video, delivered through either a stand-alone HMD or a tablet screen, elicited significant improvements in nature engagement outcomes among university students. These outcomes were associated only to a limited extent with the individual’s sense of presence perceived during the virtual nature experience. No association was found concerning cybersickness. These findings indicate that virtual nature can offer the opportunity to connect with actual nature among university students. Considering the mental health and well-being benefits associated with nature experiences, these results can inform future research design and intervention among other groups.

## Appendix

Multimedia Appendix 1 Overall questionnaires.

Multimedia Appendix 2 CONSORT 2010 checklist.

## Abbreviations

CNS: Connectedness to Nature Scale
CONSORT: Consolidated Standards of Reporting Trials
HMD: head-mounted display
INT-GE: Intention to Perform Green Exercise questionnaire
IVN: immersive virtual nature
NIVN: nonimmersive virtual nature
SSQ: Simulator Sickness Questionnaire
VR: virtual reality

## Data Availability

The datasets generated or analyzed during this study are available from the corresponding author on reasonable request.
