# Supplementary material for: Nature Engagement Outcomes of Viewing Nature Through a 360° Video or a Tablet Screen: Randomized Trial
Source: JMIR Serious Games. 2025 Jun 16;13:e63424. doi: 10.2196/63424 (PMC12209722; doi:10.2196/63424)
Supplement: Multimedia Appendix 1 [file games_v13i1e63424_app1.docx]

**Previous visits to the hiking location**

Have you been to Dronningslepet (the place you got information about) before?

Yes

No

I don’t remember

**Intention to participate in the organized hiking tour**

In the following, you will be asked a question about your intention to participate in the trip to Dronningslepet (the place you got information about)

**Choose the number that matches how much you agree with the statements.**

After I received information about the organized trip to Dronningslepet, the probability that I will participate in the tour is…

| **Very low** |  |  |  | **Neither  low/high** | | |  |  |  | **Very high** |
| --- | --- | --- | --- | --- | --- | --- | --- | --- | --- | --- |
| **0** | **1** | **2** | **3** | **4** | **5** | **6** | **7** | **8** | **9** | **10** |

**Intention to visit the hiking location**

In the following, you will be asked a question about your intention to visit Dronningslepet (the place you got information about)

Choose the number that matches how much you agree with the statements.

After I received information about Dronningslepet, I consider visiting it within the next 5 weeks.

| **Strongly disagree** |  |  |  | | |  | **Strongly agree** | |
| --- | --- | --- | --- | --- | --- | --- | --- | --- |
| **0** | **1** | **2** | **3** | **4** | **5** | **6** | **7** |  |

I want to visit the place I was informed about within the next 5 weeks.

| **Strongly disagree** |  |  |  | | |  | **Strongly agree** | |
| --- | --- | --- | --- | --- | --- | --- | --- | --- |
| **0** | **1** | **2** | **3** | **4** | **5** | **6** | **7** |  |

The probability that I will go to Dronningslepet during the next 5 weeks is very high.

| **Strongly disagree** |  |  |  | | |  | **Strongly agree** | |
| --- | --- | --- | --- | --- | --- | --- | --- | --- |
| **0** | **1** | **2** | **3** | **4** | **5** | **6** | **7** |  |

**Connectedness to nature scale – state version**In the following, you will be asked a question about your relationship with nature
Choose the number that matches how much you agree with the statements.

To what extent do you agree with the following statements regarding how you feel RIGHT NOW?

| \| Strongly disagree \|  \|  \| Neither agree \|  \|  \| Strongly disagree \| \| --- \| --- \| --- \| --- \| --- \| --- \| --- \| \| 1 \| 2 \| 3 \| 4 \| 5 \| 6 \| 7 \| | | |
| --- | --- | --- | --- | --- | --- | --- | --- | --- | --- | --- | --- | --- | --- | --- | --- | --- |
| **1.** | Right now I’m feeling a sense of oneness with the natural world around me. | ________ |
| **2.** | At the moment, I’m feeling that the natural world is a community to which I belong. | ________ |
| **3.** | I presently recognize and appreciate the intelligence of other living organisms. | ________ |
| **4.** | At the present moment, I don’t feel connected to nature. | ________ |
| **5.** | At the moment, I can imagine myself as part of the larger cyclical process of living. | ________ |
| **6.** | At this moment, I’m feeling a kinship with animals and plants. | ________ |
| **7.** | Right now, I feel as though I belong to the earth just as much as it belongs to me. | ________ |
| **8.** | Right now, I am feeling deeply aware of how my actions affect the natural world. | ________ |
| **9.** | Presently, I feel like I am part of the web of life. | ________ |
| **10.** | Right now, I feel that all inhabitants of earth, human and nonhuman, share a common life force. | ________ |
| **11.** | At the moment, I am feeling embedded within the broader natural world, like a tree in a forest. | ________ |
| **12.** | When I think of humans’ place on earth right now, I consider them to be the most valuable species in nature. | ________ |
| **13.** | At this moment, I am feeling like I am only a part of the natural world around me, and that I am no more important than the grass on the ground or the birds in the trees. | ________ |

**Intention to perform green exercise**

In the following, you will be asked a question about your intention to do "green exercise" over the next 5 weeks

Choose the number that matches how much you agree with the statements.

Examples of what is meant by "green exercise": Walking or training in a park, green or nature areas. It can also be walking or cycling to and from work, walking or exercising with a dog or other pet, etc.

I expect to do Green Exercise

| **Strongly disagree** |  |  |  | | |  | **Strongly agree** | |
| --- | --- | --- | --- | --- | --- | --- | --- | --- |
| **0** | **1** | **2** | **3** | **4** | **5** | **6** | **7** |  |

I want to do Green Exercise

| **Strongly disagree** |  |  |  | | |  | **Strongly agree** | |
| --- | --- | --- | --- | --- | --- | --- | --- | --- |
| **0** | **1** | **2** | **3** | **4** | **5** | **6** | **7** |  |

The likelihood of me doing Green Exercise is...

| **Strongly disagree** |  |  |  | | |  | **Strongly agree** | |
| --- | --- | --- | --- | --- | --- | --- | --- | --- |
| **0** | **1** | **2** | **3** | **4** | **5** | **6** | **7** |  |

I plan to do Green Exercise

| **Strongly disagree** |  |  |  | | |  | **Strongly agree** | |
| --- | --- | --- | --- | --- | --- | --- | --- | --- |
| **0** | **1** | **2** | **3** | **4** | **5** | **6** | **7** |  |

I intend to do Green Exercise

| **Strongly disagree** |  |  |  | | |  | **Strongly agree** | |
| --- | --- | --- | --- | --- | --- | --- | --- | --- |
| **0** | **1** | **2** | **3** | **4** | **5** | **6** | **7** |  |

**Green exercise routine**

*In the following, you will be asked a question about how often you engage in "green exercise"*
Choose the option that suits you.

How often do you do "green exercise"?

More than 3 times a week

2 times a week

1 time a week
 Less than 1 time a week

**Presence**

In the following, you will be asked questions about how you felt while watching the video in VR

Choose the number that matches how much you agree with the statements.

| \| **Absolutely agree** \|  \|  \|  \| **Neither agree/disagree** \| \| \|  \|  \|  \| **Absolutely disagree** \| \| --- \| --- \| --- \| --- \| --- \| --- \| --- \| --- \| --- \| --- \| --- \| \| **0** \| **1** \| **2** \| **3** \| **4** \| **5** \| **6** \| **7** \| **8** \| **9** \| **10** \|  \| 1. In the virtual reality world I had the sense of ‘being there’ \|  \| \| --- \| --- \| \| 1. I thought of the virtual environment as equal to the real environment \|  \| \| 1. The virtual world became more real or present to me compared to the real world. NB: by ‘real world’ we mean the room where you were undergoing the test \|  \| \| 1. During the VR video, I often thought of the other person(s) in the room with me \|  \| \| 1. It would have been more enjoyable to engage with the ‘virtual world’ with no-one else in the room \|  \| \| 1. Whilst I was watching the VR video, I paid much attention to other noises around me in the room \|  \| \| 1. The virtual world appeared flat and missing in depth \|  \| |
| --- | --- | --- | --- | --- | --- | --- | --- | --- | --- | --- | --- | --- | --- | --- | --- | --- | --- | --- | --- | --- | --- | --- | --- | --- | --- | --- | --- | --- | --- | --- | --- | --- | --- | --- | --- | --- |
|  |

**Simulator-sickness questionnaire**

In the following, you will be asked questions about how you felt while watching the VR video

Choose the option that suits you.

To what extent are you experiencing the following symptoms right now?

| 1. General discomfort | Not at all | A little | Moderately | A lot |
| --- | --- | --- | --- | --- |
| 2. Fatigue | Not at all | A little | Moderately | A lot |
| 3. Headache | Not at all | A little | Moderately | A lot |
| 4. Eyestrain | Not at all | A little | Moderately | A lot |
| 5. Difficulty focusing | Not at all | A little | Moderately | A lot |
| 6. Increased salivation | Not at all | A little | Moderately | A lot |
| 7. Sweating | Not at all | A little | Moderately | A lot |
| 8. Nausea | Not at all | A little | Moderately | A lot |
| 9. Difficulty concentrating | Not at all | A little | Moderately | A lot |
| 10. Fullness of head | Not at all | A little | Moderately | A lot |
| 11. Blurred vision | Not at all | A little | Moderately | A lot |
| 12. Dizzy (eyes open) | Not at all | A little | Moderately | A lot |
| 13. Dizzy (eyes closed) | Not at all | A little | Moderately | A lot |
| 14. Vertigo | Not at all | A little | Moderately | A lot |
| 15. Stomach awareness | Not at all | A little | Moderately | A lot |
| 16. Burping | Not at all | A little | Moderately | A lot |
